# Reducing AsA Leads to Leaf Lesion and Defence Response in Knock-Down of the AsA Biosynthetic Enzyme GDP-D-Mannose Pyrophosphorylase Gene in Tomato Plant

**DOI:** 10.1371/journal.pone.0061987

**Published:** 2013-04-23

**Authors:** Chanjuan Zhang, Bo Ouyang, Changxian Yang, Xiaohui Zhang, Hui Liu, Yuyang Zhang, Junhong Zhang, Hanxia Li, Zhibiao Ye

**Affiliations:** The Key Laboratory of Horticultural Plant Biology, Ministry of Education, Huazhong Agricultural University, Wuhan, China; Nanjing Agricultural University, China

## Abstract

As a vital antioxidant, L-ascorbic acid (AsA) affects diverse biological processes in higher plants. Lack of AsA in cell impairs plant development. In the present study, we manipulated a gene of GDP-mannose pyrophosphorylase which catalyzes the conversion of D-mannose-1-P to GDP-D-mannose in AsA biosynthetic pathway and found out the phenotype alteration of tomato. In the tomato genome, there are four members of *GMP* gene family and they constitutively expressed in various tissues in distinct expression patterns. As expected, over-expression of *SlGMP3* increased total AsA contents and enhanced the tolerance to oxidative stress in tomato. On the contrary, knock-down of *SlGMP3* significantly decreased AsA contents below the threshold level and altered the phenotype of tomato plants with lesions and further senescence. Further analysis indicated the causes for this symptom could result from failing to instantly deplete the reactive oxygen species (ROS) as decline of free radical scavenging activity. More ROS accumulated in the leaves and then triggered expressions of defence-related genes and mimic symptom occurred on the leaves similar to hypersensitive responses against pathogens. Consequently, the photosynthesis of leaves was dramatically fallen. These results suggested the vital roles of AsA as an antioxidant in leaf function and defence response of tomato.

## Introduction

In higher plants, reactive oxygen species (ROS) are produced as by-products in most energy-generating processes, such as photosynthesis and respiration. An electron reduction of O_2_ leads to the formation of superoxide radical (O_2_^−^), which is then disproportionated by superoxide dismutase (SOD) to O_2_ and hydrogen peroxide (H_2_O_2_). ROS production in plant cells is low under optimal growth conditions, but increases dramatically when plants are subjected to abiotic stresses and pathogen attack. Unfavourable environmental conditions, such as cold, heat, drought, and salt, limit the rate of carbon fixation, which results in an increase in photoinhibition and overproduction of superoxide radicals and H_2_O_2_ [1]. Furthermore, oxidative burst is one of the most rapid defence reactions to pathogen attack, which changes the production of superoxide and H_2_O_2_ at the infection site [2].

Excessive ROS can induce programmed cell death and necrosis [3]. In higher plants, the levels of ROS are strictly regulated by an efficient battery of enzymatic and non-enzymatic antioxidants [2]. Among them, ascorbic acid (AsA) acts as one of the most abundant antioxidants against oxidative stress [4]. In plants, chloroplasts are potentially the major site for the generation of ROS, in which AsA is present at a high level, at concentration of 20 mM or more. Thus, AsA plays a central function in photoprotection, including scavenger of ROS generated by photosynthesis and respiration, cofactor for violaxanthin deepoxidase, and photosystem II electron donor [5].

**Figure 1:**
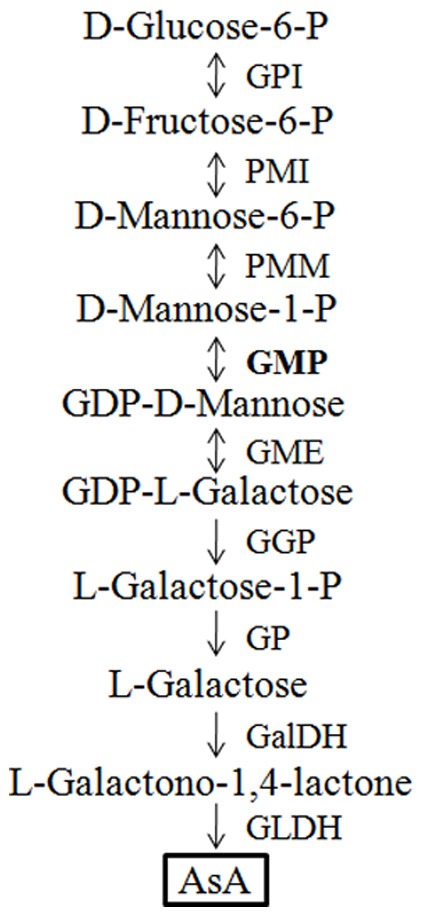
Major AsA biosynthetic pathway in higher plants (Smirnoff-Wheeler ’**s pathway).** GPI, glucose-6-phosphate isomerase; PMI, phosphomannose isomerase; PMM, phosphomannomutase; GMP (marked in bold), GDP-D-mannose pyrophosphorylae; GME, GDP-D-mannose-3′,5′-epimerase; GGP, GDP-L-galactose-1-phosphate phosphorylase; GP, L-galactose-1-phosphate phosphatase; GalDH, L-galactose dehydrogenase; GLDH, L-galactono-1,4-lactone dehydrogenase.

Although alternative biosynthetic pathways have been proposed [6]–[8], Smirnoff-Wheeler’s pathway [9] (Fig. 1) has been proved to be the major functional pathway by biochemical and genetic approach. GDP-D-mannose pyrophosphorylase (GMP) catalyzes the conversion of D-Mannose-1-P to GDP-D-Mannose, an initial step in the Smirnoff-Wheeler’s pathway [9]. The importance of GMP in the control of AsA biosynthesis has been confirmed in some plants. The significant reduction of AsA in the *vtc1* mutant of *Arabidopsis* was caused by a point mutation in *GMP* gene [10]. Antisense inhibition of *GMP* gene in the transgenic potato (*Solanum tuberosum*) plants reduced AsA contents both in leaves and tubers [11]. In acerola (*Malpighia glabra*), the *MgGMP* gene expression displayed a strong correlation with the AsA contents in the ripening fruit [12]. In addition, over-expression of *MgGMP* gene increased AsA content by approximately two-fold in tobacco (*Nicotiana tabacum*) [13].

Tomato is a representative of edible fruit plants with an abundance of AsA. Recently, several progresses about AsA metabolism in tomato have been achieved [14]–[19]. Genes involved in tomato AsA biosynthesis have been largely identified [20]–[24], and some of them have been functionally characterized, such as L-galactono-1,4-lactone dehydrogenase (*GLDH*) [20] and GDP-D-mannose 3′, 5′-epimerase (*GME*) [21], [22]. In tomato genome, GMP has four isoforms (GMP1-GMP4). Currently, some progresses have been made in understanding the biological function of tomato *GMP* genes, especially *GMP3*. Expression profiling analysis revealed that *SlGMP3* mRNA accumulated during the early stages of tomato fruit development and was down-regulated in mature green fruits subjected to ethylene and various stresses [16]. In addition, over-expression of *SlGMP3* gene in tobacco significantly increased AsA content in leaf and improved the tolerance to high and low temperature stress by enhancing antioxidation capacity [25]. However, whether *SlGMP3* affects the AsA biosynthesis and accumulation in tomato remains unclear.

To better understand the regulation of AsA biosynthesis in tomato, we generated *SlGMP3* over-expressing and knocked-down transgenic tomato plants, and analyzed the subsequent effects on AsA metabolism and plant growth. Results indicated that elevated *GMP3* expression increased total AsA levels in tomato leaves and fruits, and then enhanced photo-oxidative stress tolerance, whereas RNAi silencing of GMP in tomato could effectively reduce AsA contents, leading to ROS accumulation with leaf lesion formation and senescence, and activation of defence responses. These imply the vital role of AsA as an antioxidant in tomato.

## Materials and Methods

### Plant Materials

Tomato (*Solanum lycopersicum* cv. Ailsa Craig, AC) plants were grown in a naturally illuminated glasshouse. Tissues from roots, stems, leaves, flowers, and fruits at various developmental stages of AC plants were collected, immediately frozen in liquid nitrogen, and stored at −80°C until use.

### Gene Cloning, Vector Construction and Tomato Transformation

The 1236 bp fragment, including the full open reading frame of *SlGMP3* (SGN-U568547), was amplified using the primer sets of GMP3F and GMP3R (Table S1), and then cloned into pMD18-T vector (Takara, Japan). Subsequently, over-expression and RNAi vectors were constructed by subcloning the full length cDNA of *SlGMP3* into pMV and pHGRV vectors, respectively, under the control of CaMV35S promoter. The constructs were introduced into *Agrobacterium tumefaciens* strain EHA105 by electroporation. Tomato seeds of Ailsa Craig were used for transformation, performed according to the methods described by Fillatti et al. [26]. Independent transgenic plants were confirmed by polymerase chain reaction (PCR) using primer combinations between CaMV35S promoter specific primers CaMV35SF or gate35SF and *SlGMP3* gene specific primer GMP3R (Table S1) and Southern blot. The transcript levels of *SlGMP3* as well as other three members of *SlGMP* gene family in the transgenic lines were analyzed via semi-quantitative reverse transcriptase (RT)-PCR and real-time RT-PCR.

### Semi-quantitative and Real-time RT-PCR Analysis

Total RNA was isolated using TRIzol® reagent (Introvigen, USA), and DNase I was used to clean out DNA before reverse transcription. The reverse transcript reaction was performed with MMLV reverse transcriptase (Toyobo, Osaka, Japan) following the manufacturer’s protocol. Reverse transcript products were used as template for semi-quantitative RT-PCR and real-time RT-PCR. All reaction was assayed in triplicates. Semi-quantitative RT-PCR was performed to preliminarily analyze the expression levels of *SlGMP3* in the transgenic lines. It was carried out using the following program: an initial denaturation of 94°C for 3 min, followed by 24–28 circles of 94°C for 30 s, 56°C for 30 s, and 72°C for 1 min, and a final extension at 72°C for 10 min. PCR products were detected by 1% agarose gel in 1×TAE with EtBr. Real-time RT-PCR was performed using LightCycler 480 SYBR Green I Master kit (Roche, Germany) according to the supplier’s instruction to analyze the expression levels of the genes involved in AsA biosynthesis, photosynthesis, antioxidant system, and defence response in the transgenic tomato plants. Real-time RT-PCR amplification step consisted of a pre-incubation at 95°C for 5 min, followed by 40 circles of 95°C for 10 s, 60°C for 15 s and 72°C for 20 s. PCR products were monitored by the LightCycler 480 Real-Time PCR Detection System. Tomato β-actin gene was used as a reference gene to optimize semi-quantitative RT-PCR and as internal standard in real-time RT-PCR. The primers used are listed in Tables S1, S2, S3 and S4.

### Oxidative Stress Treatment

One-month-old plantlets of *SlGMP3* over-expressing lines and wild type grown in compost plastic pots in a naturally illuminated glasshouse were sprayed with 75 µM methyl viologen (MV) solution or distilled water (control) for 2 d. The MV dissolved in 0.1% Tween-20 solution (100 mL) was applied to six plants. The fourth leaf samples were taken at 7 d post-treatment. The effect of oxidative stress on *SlGMP3* over-expressing transgenic plants was assessed by measuring the chlorophyll and malondialdehyde (MDA) contents in the leaves. The experiment was replicated three times.

### Measurement of Total Ascorbate

The AsA content was determined using high-performance liquid chromatography as described by Rizzolo et al. [27]. Briefly, samples were ground under liquid nitrogen and homogenised in 5 mL of cold 0.1% (w/v) metaphosphoric acid. The homogenate was centrifuged at 12,000 g for 10 min at 4°C. Then the supernatant was filtered through a Millipore membrane (0.22 µm). An aliquot of 300 µL was incubated with 300 µL 50 mM dithiothreitol for 15 min at room temperature. Then, the extracts were analysed by HPLC using an SB-aq column (Agilent) eluted with acetate buffer (0.2 mol/L, pH 4.5) at a flow rate of 1.0 mL/min to measure total ascorbate. Elutes were detected at 254 nm, and a standard curve from 2 to 40 µg/mL AsA was obtained.

### Determination of Chlorophyll, MDA, and Net Photosynthesis Rate

Chlorophyll content was determined by grinding leaf tissues under liquid nitrogen and extracting with 80% (v/v) acetone under low light intensity using the procedure described by Wellburn’s [28]. MDA was assayed for indirect evaluation of lipid peroxidation using trichloroacetic acid, as described previously by Heath and Parker [29]. Net photosynthesis rate was measured using a portable photosynthetic system (CIRAS-2, PP System, USA) according to the supplier’s manual.

### Histochemistry

Leaf samples taken from two-month-old tomato plants were stained with trypan blue and 3,-3′-diaminobenzidine (DAB) solution to visualize dead cells and detect the presence of H_2_O_2_, respectively. Trypan blue staining was performed as previously described [30]. Leaves were submerged in a 70°C LPTB solution [2.5 mg/mL trypan blue, 25% (w/v) lactic acid, 23% water-saturated phenol, 25% glycerol, and H_2_O], vacuum infiltrated for 5 min, and then repeated one time. Subsequently, the samples were heated over boiling water for 2 min and cooled for 1 h. The LPTB solution was then replaced with a chloral hydrate solution (25 g in 10 mL H_2_O) for destaining. After multiple exchanges, the samples were equilibrated in 70% glycerol and photographed. H_2_O_2_ was visually detected in the leaves of tomato plants using DAB as the substrate [31]. Briefly, the leaves were cleaned and placed in 1 mg/mL DAB, pH 3.8, under light at 25°C for 8 h. The experiment was terminated by immersing the leaves in boiling 96% ethanol for 10 min. After cooling, the leaves were placed in fresh 96% ethanol for 4 h at room temperature and photographed. The deep brown polymerization product was produced via the reaction of DAB with H_2_O_2_.

### Statistic Analysis

Data analysis was performed using SAS software, and significant differences were calculated using the Student’s *t*-test at 95% confidence limit.

## Results

### Expression Patterns of *GMP* Genes in Tomato

**Figure 2:**
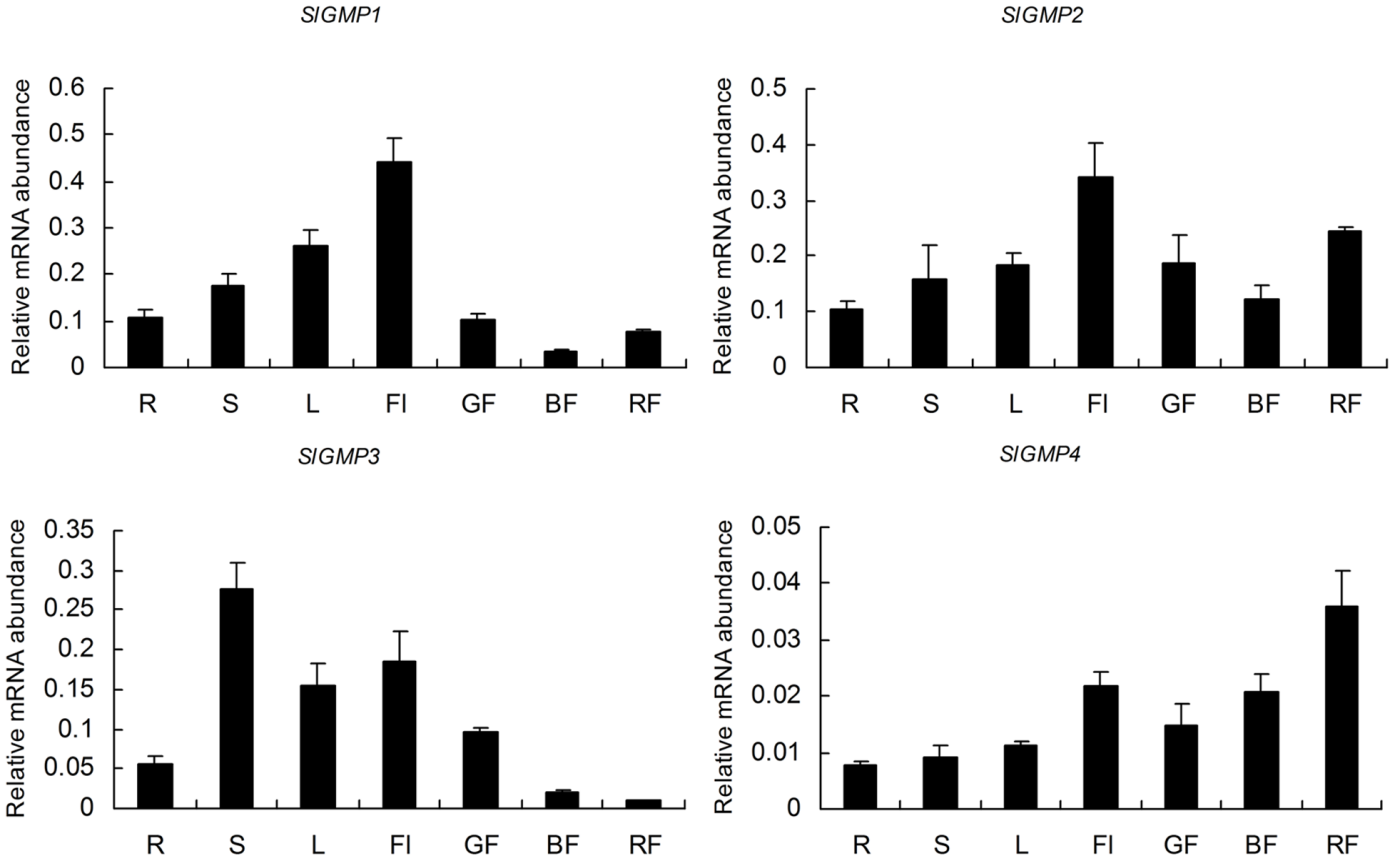
Relative expression analysis of four members of *SlGMP* gene family in various tissues of tomato variety Ailsa Craig. R: root, S: stem, L: leaf, Fl: flower, GF: green fruit, BF: breaker fruit, and RF: red fruit. Data obtained by real-time RT-PCR were normalized against *Actin*.

Four unigenes corresponding to the amino acid sequences of GMP (*GMP1*, SGN-U563807; *GMP2*, SGN-U568548; *GMP3*, SGN-U568547; and *GMP4*, SGN-U584300) exist in tomato genome as reported by Massot et al. [17]. Blast results showed that *GMP1* and *GMP3* are both located on chromosome 3, and *GMP2* and *GMP4* are located on chromosome 6 and 9, respectively. The full-length cDNA of *SlGMP3* was isolated previously by our group [23]. We investigated the spatial and temporal expression patterns of four *GMP* members via real-time RT-PCR analysis. All of them were expressed constitutively in the tissues tested though distinct expression patterns (Fig. 2). For *SlGMP3*, the transcript levels were high in stems, flowers, and young leaves, whereas low in roots, and fruits at breaker and red ripe stages (Fig. 2). The expressions of the other members, *SlGMP1* and *SlGMP2*, followed in a similar pattern, which were high in flowers, low in roots and breaker fruits, and slightly increased at the red ripe stage (Fig. 2). For *SlGMP4*, the expression was extremely distinct at low in most tissues, especially in vegetative tissues (Fig. 2).

### Identification of Tomato Transgenic Plants

**Figure 3:**
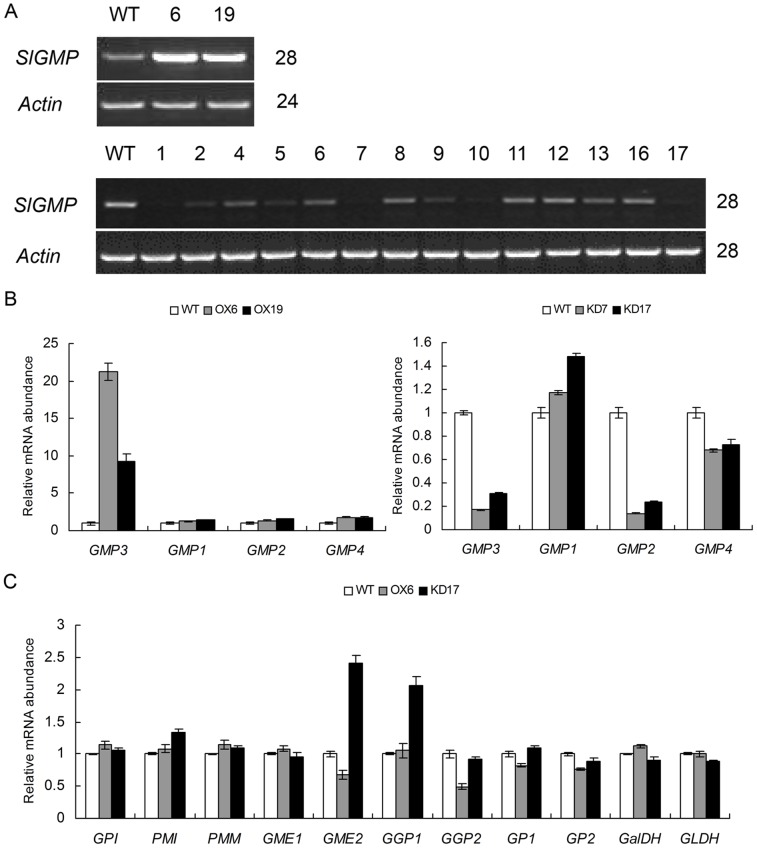
Expression analysis of four *SlGMPs* and other AsA biosynthesis-related genes in *SlGMP3* transgenic plants. (A) RT-PCR analysis of *SlGMP3* expression in the young leaves of two *SlGMP3*-OX lines (upper panel) and 17 *SlGMP3* RNAi lines (bottom panel). The PCR circle numbers are indicated on the right. (B) Relative expression analysis of four members of *SlGMP* gene family in the young leaves of lines OX6 and OX19 (left) and lines KD7 and KD17 (right) via real-time RT-PCR. (C) Relative expression analysis of AsA biosynthesis-related genes in the young leaves of lines OX6 and KD17 via real-time RT-PCR. Data were obtained by normalizing against *Actin* and shown as a percentage of wild-type plants.

Thirty-eight *SlGMP3* over-expressing (OX) and seventeen RNAi (KD) transgenic plants were obtained and confirmed by PCR using genomic DNA as template and 35S forward and gene-specific reverse primers. The expression level of *SlGMP3* gene in young leaves of transgenic as well as wild-type plants was examined by semi-quantitative RT-PCR (Fig. 3A) and real-time RT-PCR (Fig. 3B). Two over-expressing lines (OX6 and OX19) and two RNAi lines (KD7 and KD17) with significant changes were selected for further study.

In order to make clear whether other three members of *SlGMP* gene family were affected in the *SlGMP3* transgenic plants, we investigated their expressions in young leaves (Fig. 3B). In two *SlGMP3* over-expressing lines, the expression of *SlGMP1*, *SlGMP2* and *SlGMP4* was not significantly affected. However, in *SlGMP3* RNAi lines, only *SlGMP2* was markedly down-regulated. This is due to the fact that *SlGMP2* has 86% homology to *SlGMP3* with ten > = 20 base pair length identity (Fig. S1), while *SlGMP1* and *SlGMP4* share low identity (only 23% and 40%, respectively) with *SlGMP3*. Due to repression of both *SlGMP2* and *SlGMP3*, *SlGMP3* RNAi lines were designated as *SlGMP2/3*-KD lines in the following.

### Alteration of AsA Pool Size in Transgenic Tomato Plants

**Figure 4:**
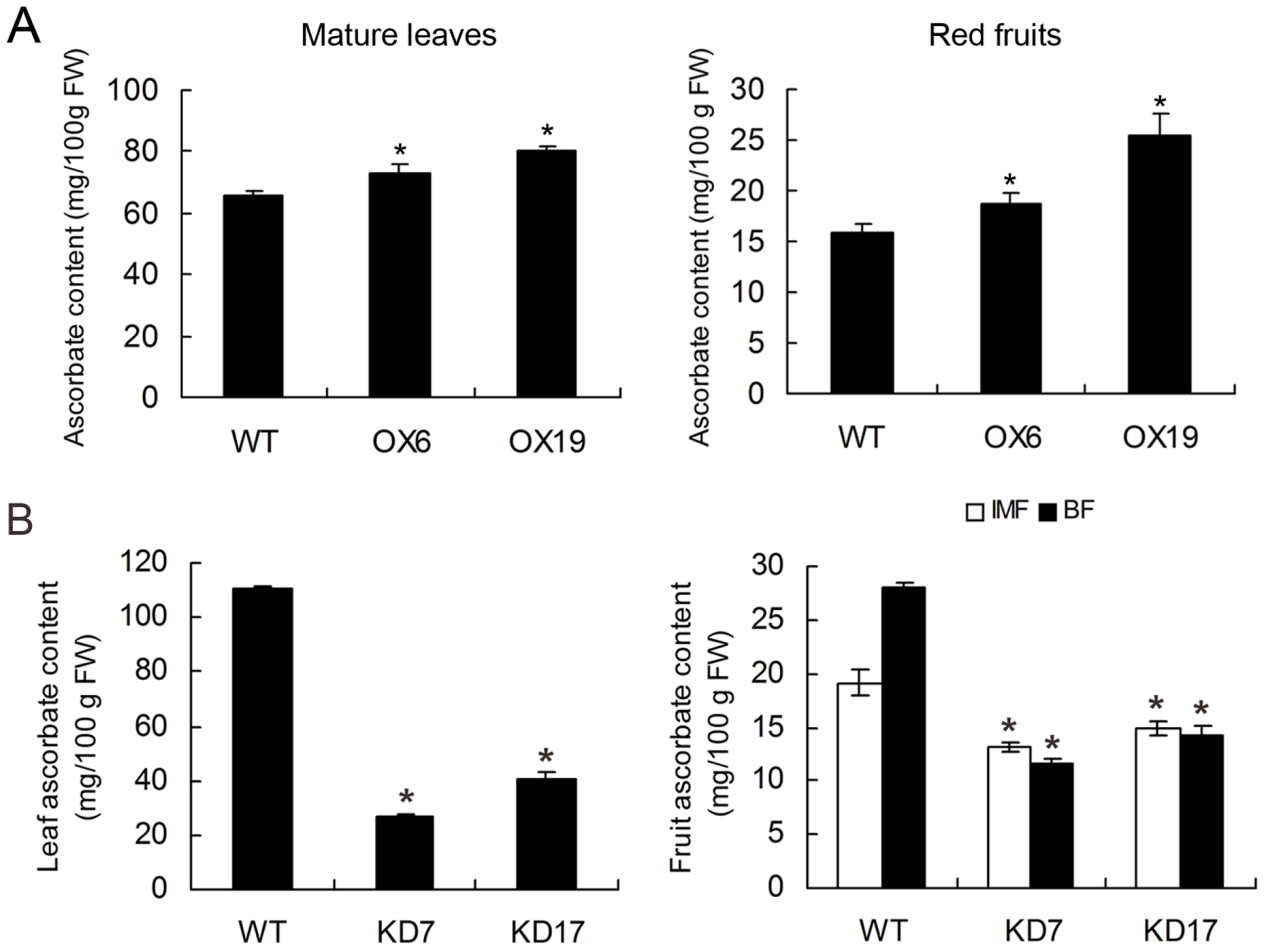
AsA content analysis in *SlGMP3* transgenic plants. (A) Total AsA contents in the mature leaves (left) and red fruits (right) of lines OX6 and OX19. (B) Total AsA contents in the mature leaves (left) and immature green fruits (IMF) and breaker fruits (BF) (right) of lines KD7 and KD17. As the experiments of (A) and (B) were carried at two separate times, there were some differences in AsA contents in leaves of the wild-type tomato plants. Data are presented as mean ± SD of four independent plants per line. Asterisk indicates significant differences from the control (*P*>0.95).

The total AsA contents of both *SlGMP3*-OX and *SlGMP2/3*-KD lines were altered compared to the non-transgenic plants. Constitutive over-expression of *SlGMP3* increased the total AsA contents in the fully expanded leaves and red ripe fruits (Fig. 4A). However, in *SlGMP2/3*-KD lines, the total AsA contents in leaves, immature green fruits, and breaker fruits decreased dramatically (Fig. 4B). With the developments of fruit, the AsA levels increased up to 46% in breaker stage compared with immature green stage in wild-type plants, whereas no major difference was observed between two stages of *SlGMP2/3*-KD lines (Fig. 4B). Moreover, a correlation between AsA content and the suppression extent of *SlGMP* genes was observed in KD7 and KD17 lines. These results indicate that over-expressing *SlGMP3* could enhance AsA accumulation, and knock-down of *SlGMP3* with *SlGMP2* significantly affects the total AsA pool size in tomato leaves and fruits.

The expression levels of other genes in the Smirnoff-Wheeler’s pathway were also examined in transgenic and wild-type plants. The results showed the regulation of AsA biosynthesis altered in leaves. The transcript abundances of biosynthetic genes *GME* and GDP-L-galactose-1-phosphate phosphorylase (*GGP*) were remarkably affected. Over-expression of *SlGMP3* down-regulated *SlGME2* and *SlGGP2* (reduced nearly 50%) (Fig. 3C). On the contrary, in *SlGMP2/3*-KD transgenic line KD17, *SlGME2* and *SlGGP1* were significantly up-regulated (over one fold) compared with the wild-type plants (Fig. 3C). Most other genes of AsA biosynthetic pathway remained unchanged in transgenic plants.

### Enhanced Tolerance to Oxidative Stress through Over-expressing *SlGMP3* in Tomato

**Figure 5:**
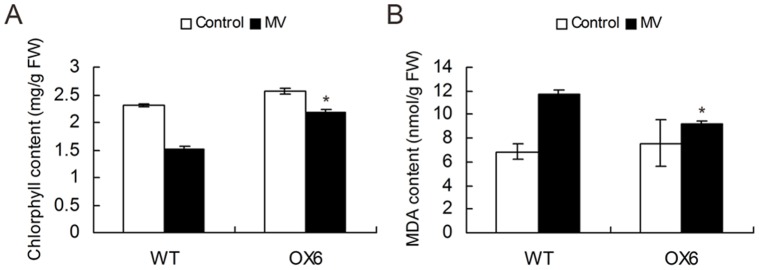
Improvement of the photo-oxidative stress tolerance in tomato plants via over-expressing *SlGMP3*. Chlorophyll content (A) and MDA content (B) in the fourth leaf of the MV-treated and untreated plants measured at 7 d post-treatment. Data are presented as mean ± SD (N = 6) from triplicate independent measurements. Asterisk indicates significant differences from the control (*P*>0.95).

To evaluate whether the over-expression of *SlGMP3* could protect transgenic plants against oxidative damage, MV was used to induce oxidative stress and sprayed on one-month-old plantlets of line OX6 and wild type. After the treatment, we found less chlorotic spots on the leaves of line OX6 than wild type. The oxidative damage caused by MV was examined through the extents of leaf chlorophyll loss and MDA (membrane-lipid peroxidation product) increase. Under normal conditions, chlorophyll content was not significantly different between wild-type and transgenic plants. However, after MV treatment, the chlorophyll content in the wild-type plants decreased by 34%, whereas no significant change in line OX6 (Fig. 5A). And the same as the MDA content in wild-type plants increased up to 70%, whereas only 21% increase was found in line OX6 (Fig. 5B). These results indicate that over-expressing *SlGMP3* improves the tolerance of the plants to oxidative stress.

### Phenotypic Alteration in *SlGMP2/3*-KD Plants

**Figure 6:**
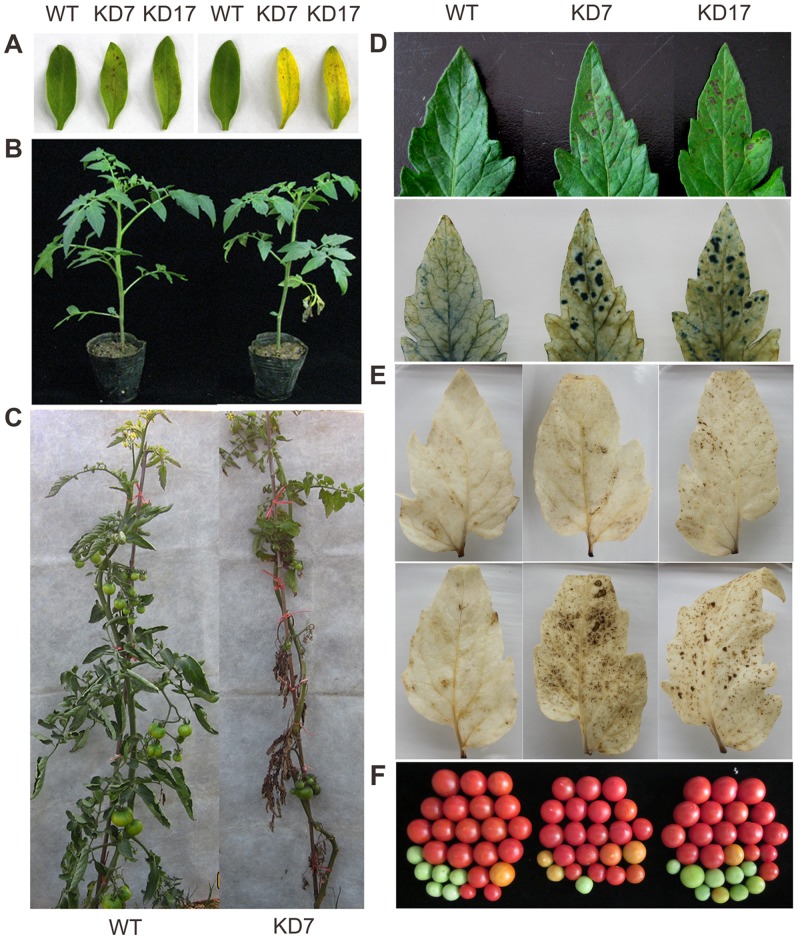
Phenotype comparisons of *SlGMP2/3*-KD and wild-type plants. (A) Altered cotyledon morphology of *SlGMP2/3*-KD vs wild-type plants. KD7 and KD17 cotyledons developed lesions (left) at three weeks post germination and accelerated senescence (right) compared with the wild type. (B) Seedlings of wild-type (left) and KD7 (right) plants. The bottom leaves of KD7 plant started to wilt. (C) Plant morphology of the three-month-old wild-type (left) and KD7 (right) plants. Middle and bottom leaves of KD7 plants became dry wilted. (D) Lesion formation on KD7 and KD17 leaves. The leaf lesion areas on the two-month-old plants of KD7 and KD17 (upper panel) are consistent with the areas of dead cells revealed through trypan blue staining (bottom panel). (E) H_2_O_2_ accumulation in the leaves of KD7 and KD17. H_2_O_2_ accumulation was revealed via DAB staining in leaves without (upper panel) and with (lower panel) visible lesions from the two-month-old plants of KD7 and KD17. (F) Tomato fruits harvested from four-month-old wild-type and transgenic plants.

The *SlGMP3*-OX plants grew normally during the whole life circle. On the contrary, the *SlGMP2/3*-KD plants exhibited a lesion-mimic phenotype from the very early stage of seedlings, and the lesions spontaneously developed on leaves. Some lesions initially appeared on the cotyledon surfaces at three weeks post germination; the cotyledons turned yellow (Fig. 6A) and then dropped off the plants. Subsequently, lesions occurred on the lower true leaves (Fig. 6B), and spread from the bottom to the upper leaves (Fig. 6C). The necrotic lesions started from the leaflet tips of the compound leaves (Fig. 6D), and developed in a typical circular pattern and spread until the entire leaves turned chlorotic, and finally fell. The cell deaths of the lesioned leaves were observed via trypan blue staining. The dead cells were consistent with macroscopic lesions before staining in the leaves of lines KD7 and KD17, whereas no staining was observed in wild-type leaves (Fig. 6D). The mature leaves of lines KD7 and KD17 withered faster than the wild type. With plants growing up to the reproductive stage, most leaves of line KD7 were dried out, whereas the wild-type plants remained green (Fig. 6C). We noticed the occurrence of lesions was more severe in line KD7 than line KD17. It indicates that phenotypic changes are correlated with the inhibition extent of *SlGMP* genes (Fig. 3B) in tomato.

Although the flowering time and the yield of *SlGMP2/3*-KD plants were not influenced, the fruit weight was significantly decreased in line KD7 (Fig. 6F and Table S5).

### Oxidative Burst in the Leaves of *SlGMP2/3*-KD Plants

The spontaneous lesions on the leaves of *SlGMP2/3*-KD plants developed in a similar manner as a hypersensitive response after pathogen attack. Oxidative burst, wherein large quantities of ROS are generated, is an early plant response to microbial pathogen attack. Both types of leaves with and without lesions were analyzed via DAB staining to check whether the lesions observed in the leaves of *SlGMP2/3*-KD plants were caused by H_2_O_2_ accumulation. The lesioned leaves stained more, whereas the wild type did not (Fig. 6E), showing a close relationship between lesion and H_2_O_2_ levels in tomato leaves.

**Figure 7:**
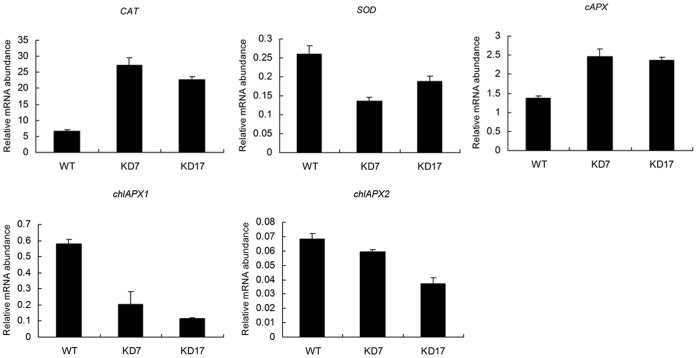
Relative transcript levels of the oxidative stress-related genes in the tomato lesioned leaves. The expression levels of oxidative related genes *CAT*, *SOD*, *cAPX*, and *chlAPXs* in the tomato lesioned leaves were measured by real-time RT-PCR. Data were normalized against *Actin*.

To examine whether the accumulation of high ROS concentrations affected the antioxidant defence system of plants, the transcript levels of several genes involved in detoxifying H_2_O_2_ or O^2−^ were measured, including catalase (*CAT*), *SOD*, and ascorbate peroxidase (*APX*) using real-time RT-PCR. We found that *SOD* and chloroplastic *APX* genes were down-regulated in lesioned leaves of lines KD7 and KD17, whereas both their *CAT* and cytosolic *APX* genes were significantly up-regulated, especially for *CAT*, which increased over two-fold compared with the wild type (Fig. 7). These results imply that the partial antioxidant defence system is triggered in response to the oxidative burst in *SlGMP2/3*-KD leaves.

### Activation of Defence Response in *SlGMP2/3*-KD Plants

**Figure 8:**
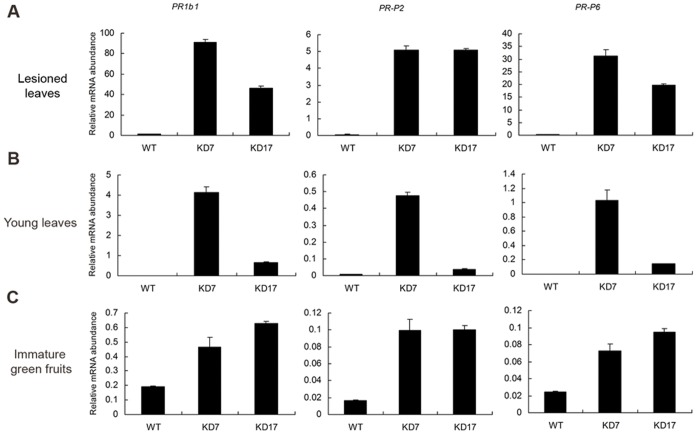
Relative transcript levels of the pathogenesis-related genes in the tomato lesioned and normal leaves and fruits. Expression analysis of the pathogenesis-related genes *PR1b1*, *PR-P2*, and *PR-P6* was performed in necrotic mature leaves (A), non-necrotic normal young leaves (B) and immature green fruits (C) of lines KD7 and KD17 by real-time RT-PCR. Data were normalized against *Actin*.

Many mutants with such lesions in plants have shown an enhanced systemic resistance to microbial pathogens. To reveal whether *SlGMP2/3*-KD plants activated pathogen defence response, the expressions of three pathogenesis-related (PR) genes, including *PR1b1* [32], *PR-P2* [33], and *PR-P6* [34], [35], were analyzed by real-time RT-PCR. These genes are known to be strongly induced locally in tomato-pathogen interactions. The results showed that the transcripts of *PR1b1*, *PR-P2*, and *PR-P6* were more abundant in lesioned leaves of lines KD7 and KD17 than those in the wild type (Fig. 8A). Moreover, three defence genes were also activated in young leaves without any lesion (Fig. 8B) and in immature green fruits without any occurrence of lesions (Fig. 8C) though the activation extent was less than that in lesioned leaves. In addition, as shown in Fig. 8, more transcripts of *PR1b1*, *PR-P2*, and *PR-P6* were induced in leaves than immature fruits. All these results seem that systemic acquired resistance (SAR) is activated in *SlGMP2/3*-KD plants.

### Impairment of the Photosynthetic System in *SlGMP2/3*-KD Plants

**Figure 9:**
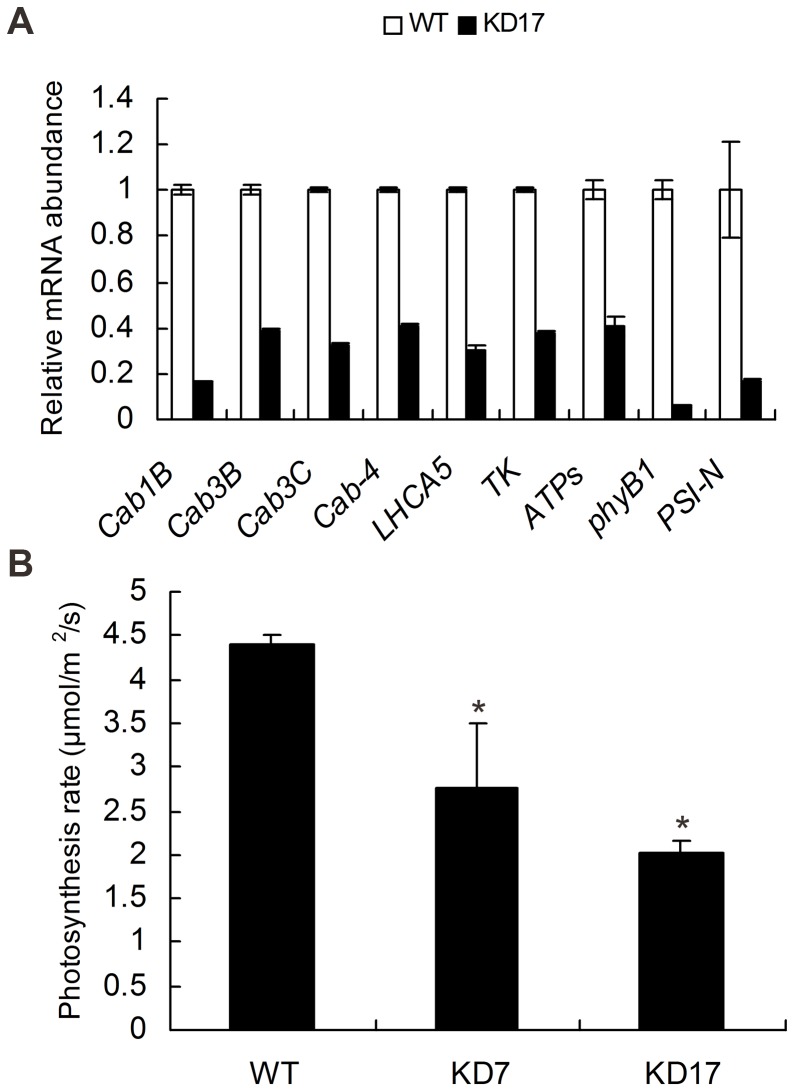
Photosynthetic system was impaired in leaves of *SlGMP2/3*-KD tomato plants. (A) Relative transcript levels of the photosynthesis-related genes in wild-type leaves and KD17 necrotic leaves were assayed via real-time RT-PCR. Data were normalized against *Actin* and shown as a percentage of wild-type plants. (B) Net photosynthesis rates of KD7 and KD17 necrotic leaves and wild-type leaves were measured using a portable photosynthetic system (CIRAS-2, PP System, USA). Data are presented as mean ± SD (N = 4) from triplicate independent measurements. Asterisk indicates significant differences from the control (P>0.95).

Primary results obtained through TOM2 Oligo chip microarray showed that some photosynthesis-related genes were up-regulated in the breaker fruits of *SlGMP3*-OX line OX19, and further confirmed via real-time RT-PCR analysis. These genes are involved in chlorophyll *a*-*b* binding, light harvesting processes in photosystem II, as well as ATP and phytochrome biosyntheses. Therefore, these genes were also analyzed in *SlGMP2/3*-KD plants. The nine selected photosynthesis-related genes were significantly down-regulated, and the net photosynthesis rates drastically declined in the slightly lesioned leaves of lines KD7 and KD17 (Fig. 9A, B).

## Discussion

GMP has been shown to affect AsA biosynthesis in *Arabidopsis* [10], potato [11], and acerola [12], [13]. Recently, expression of yeast-derived GMP gene in tomato was found to enhance AsA levels in leaves, green and red fruits [36], indicating biotechnological manipulation of AsA biosynthesis in tomato can be achieved through increasing GMP activity. In recent years, four GMP isoforms are found to exist in tomato genome [17]. However, until now, whether AsA biosynthesis could be regulated by manipulation of the four *SlGMP* genes in tomato is not clear yet. In 2006, due to the limited mRNA/EST sequence information in public databases, only one *GMP* gene (*SlGMP3*) was obtained from tomato by our group. The putative amino acid sequence of *SlGMP3* has high similarity with AtGMP and StGMP which have been proved to play important roles in ascorbate biosynthesis in Arabidopsis and potato, respectively. So we focused our work on *SlGMP3* gene to make clear its function in tomato. In this study, we generated transgenic tomato plants using constitutive over-expression and RNAi strategies for *SlGMP3* gene and studied the plant response to stress regarding the role of GMP in the Smirnoff-Wheeler’s pathway and AsA as a major antioxidant of the plant cell.

Over-expression of *SlGMP3* increased the total AsA content and enhanced antioxidant capacity, which was consistent with the results in tobacco expressing *SlGMP3* [25]. Since we constructed RNAi vector using the full length cDNA of *SlGMP3*, *SlGMP2* as well as *SlGMP3* were simultaneously suppressed, leading to significant reduction in AsA contents in leaves and fruits of tomato. This confirmed the importance of the Smirnoff-Wheeler’s pathway and the vital role of GMP activity in AsA biosynthesis in tomato plant. Plant AsA efflux is regulated by environmental factors and especially the light environment. Massot et al. [17] found that among the four GMP genes, *SlGMP1* and *SlGMP3* were regulated by light, whereas *SlGMP2* and *SlGMP4* were not in tomato leaves. It implies *SlGMP1* may also play an important role in controlling AsA biosynthesis in tomato. It should be mentioned that *SlGMP2* was severely suppressed in *SlGMP2/3*-KD lines as *SlGMP3*, then we don’t exclude the possibility that *SlGMP2* participates in controlling ascorbate content and causing phenotypic alteration of KD lines. Further investigation is needed to study on *SlGMP1* and *SlGMP2*.

Significant increase in transcript abundances of *SlGME2* and *SlGGP1* downstream of *SlGMP* was found in the *SlGMP2/3*-KD plants, possibly because of a biological process to maintain a relative, stable ascorbate output. Similarly, up-regulation of *SlGMP* genes and *SlGGP1* were observed in *SlGME* RNAi transgenic tomato plants [21]. The step controlled by GME was considered as an important control point in tomato AsA synthesis because it lies at the intersection of AsA synthesis and cell wall polysaccharides [21]. RNAi silencing of *SlGME1* and *SlGME2* in tomato effectively reduces AsA content, resulting in ROS accumulation, leaf bleaching and developmental defects [21], and over-expression of *SlGME2* could improve AsA accumulation in tomato leaves and red ripe fruits in our group [22]. For *GGP* gene, it encodes an enzyme that catalyzes the first committed step in AsA biosynthesis in plants and has been identified as rate limiting in *Arabidopsis* [37] and tobacco [38] leaves. It has been confirmed that over-expression of *GGP* gene from kiwifruit increased AsA up to six-fold in tomato fruits [39]. Recently, the vital role of *SlGGP1* in light-regulated AsA biosynthesis in tomato has been found [17]. We propose except *SlGME1*, *SlGME2* and *SlGMP3*, *SlGGP1* may be another good candidate for enhancing AsA accumulation in tomato. All these above underscore a complex regulation of the AsA pool size in tomato.

Lesions were formed on leaves throughout the whole life of *SlGMP2/3*-KD tomato plants. Cell death phenotype was previously found in the *Arabidopsis* mutant *vtc1* and GMP antisense transgenic potato plants lacking AsA [11], [40]. However, the cell death pattern of *SlGMP2/3*-KD tomato plants is more similar to the GMP antisense potato plants [11] than *vtc1* [40]. Interestingly, the cell death phenotype started earlier in *SlGMP2/3*-KD tomato plants than GMP antisense potato plants. Lesions were formed on tomato leaves even from seedling stage (Fig. 6A), while GMP antisense potato plants started to develop this phenotype 10 weeks after transfer to soil, which was considered that the imbalance between ROS and antioxidants due to the reduction in AsA contents changed the phenotype in the GMP antisense transgenic potato plants [11]. In this study, DAB staining revealed that higher concentration of H_2_O_2_ was involved in activating cell death program, which is distinct from the *vtc1* mutant, wherein the H_2_O_2_ content is not changed [41]. This is consistent with the case of lesion formation on tobacco leaves due to reduced levels of the H_2_O_2_-detoxifying enzyme CAT [42]. In our study, the increased AsA content improved the tolerance to photo-oxidative stress in *SlGMP3*-OX plants, which further supported the results of gene knocked-down.

CAT is the key enzyme that directly detoxifies H_2_O_2_ in plant antioxidant system. *CAT* mRNA abundance was significantly up-regulated in the lesioned leaves of *SlGMP2/3*-KD plants. The increased expression of *CAT* appeared to be a compensatory mechanism for the reduced levels of AsA under oxidative stress. However, the capacity of the antioxidant system is not largely changed in the *Arabidopsis* mutant *vtc1-1* [41], which might suggest that the low intracellular level of AsA is sufficient for scavenging ROS in *Arabidopsis*.

Salicylic acid (SA) is known to play an important role in the activation of defence responses in plants against microbial infection. In several identified lesion mimic mutants, the activation of PR genes was correlated with the accumulation of high SA levels [43], [44]. *PR1b1* and *PR-P2*, the marker genes in tomato SA-dependent defence pathway were activated in *SlGMP2/3*-KD plants. In previous study, H_2_O_2_ is considered to act upstream of SA in the signal transduction pathway [45]. We speculate that the enhanced production of H_2_O_2_ might induce defence response partly through SA response pathway in *SlGMP2/3*-KD plants. More studies are needed to further confirm this hypothesis.

To determine whether the activated defence response could enhance resistance to pathogens in *SlGMP2/3*-KD plants, five-week-old plantlets were challenged with the virulent bacterium *Pseudomonas syringae* pv. tomato DC3000 (*Pst*) according to the method described by Anderson [46]. However, the resistance of lines KD7 and KD17 to *Pst* DC3000 at 24/20°C day/night temperature (16 h/8 h) and 70% relative humidity was slightly improved but not statistically significant (data not shown). This result may be attributed to the low light intensity of the artificial climate chamber, in which the transgenic plants showed weaker phenotype of spontaneous cell death than that in greenhouse with a high light intensity.

Multiple functions for AsA in photosynthesis have been proposed [5]. AsA-deficient plants show zeaxanthin depletion and an inhibition of photosynthesis when exposed to high light [47]. Rubisco levels are significantly reduced in the *vtc1* mutant [48]. It is suggested that genes encoding the components of the electron transport system could be regulated by AsA [49]. In the chloroplast, AsA-GSH (reduced glutathione) cycle is the key pathway to protect the photosynthetic machinery by removing ROS. In *SlGMP2/3*-KD tomato lines, the expressions of several genes encoding photosynthetic enzymes were very significantly reduced as well as overall photosynthetic activity. This implies that the impairment of photosynthetic system may be due to the lower AsA level.

In conclusion, we have studied one of the AsA biosynthetic pathway steps using over-expression and RNAi strategies for *SlGMP3* gene in tomato plants. In doing so, we found the vital roles of AsA as an antioxidant in leaf senescence and defence response in tomato. The increased H_2_O_2_ level caused by low AsA levels might be responsible for the induction of the SA signal transduction pathway, which led to necrosis development and defence response activation. In addition to AsA biosynthesis, GMP is also need for synthesis of mannose, fucose and L-galactose containing polysaccharides in the cell wall. We do not exclude the possibility that cell wall defects participate in causing some of the phenotypes seen in our experiment and this will be investigated in our further study.

## Supporting Information

Figure S1 **Nucleotide sequence alignment of** ***SlGMP2*** **and** ***SlGMP3*** **genes.**
(TIF)

Table S1 **Primers used for** ***SlGMP3*** **amplification and identification of the transformants and RT-PCR.**
(DOC)

Table S2 **Primers used for real-time RT-PCR of the AsA biosynthesis-related genes.**
(DOC)

Table S3 **Primers used for real-time RT-PCR of the photosynthesis-related genes.**
(DOC)

Table S4 **Primers used for real-time RT-PCR of the pathogenesis-related genes and antioxidant enzyme genes.**
(DOC)

Table S5 **The fruit weights and yields of** ***SlGMP2/3*** **-KD lines and wild-type plants.**
(DOC)

## References

[1] Foyer CH, Noctor G (2005) Oxidant and antioxidant signalling in plants: a re-evaluation of the concept of oxidative stress in a physiological context. Plant Cell Environ 28: 1056–1071.

[2] Apel K, Hirt H (2004) Reactive oxygen species: metabolism, oxidative stress, and signal transduction. Annu Rev Plant Biol 55: 373–399. 15377225 10.1146/annurev.arplant.55.031903.141701

[3] Vranová E, Inzé D, Van Breusegem F (2002) Signal transduction during oxidative stress. J Exp Bot 53: 1227–1236. 11997371

[4] Smirnoff N (2000) Ascorbic acid: metabolism and functions of a multi-facetted molecule. Curr Opin Plant Biol 3: 229–235. 10837263

[5] Smirnoff N (2000) Ascorbate biosynthesis and function in photoprotection. Philos Trans R Soc Lond B Bio Sci 355: 1455–1464. 11127999 10.1098/rstb.2000.0706 PMC1692873

[6] Agius F, González-Lamothe R, Caballero JL, Muñoz-Blanco J, Botella MA, et al (2003) Engineering increased vitamin C levels in plants by overexpression of a D-galacturonic acid reductase. Nat Biotechnol 21: 177–181. 12524550 10.1038/nbt777

[7] Lorence A, Chevone BI, Mendes P, Nessler CL (2004) myo-Inositol oxygenase offers a possible entry point into plant ascorbate biosynthesis. Plant Physiol 134: 1200–1205. 14976233 10.1104/pp.103.033936 PMC389944

[8] Wolucka BA, Van Montagu M (2003) GDP-mannose 3′, 5′-epimerase forms GDP-L-gulose, a putative intermediate for the *de novo* biosynthesis of vitamin C in plants. J Biol Chem 278: 47483–47490. 12954627 10.1074/jbc.M309135200

[9] Wheeler GL, Jones MA, Smirnoff N (1998) The biosynthetic pathway of vitamin C in higher plants. Nature 393: 365–369. 9620799 10.1038/30728

[10] Conklin PL, Norris SR, Wheeler GL, Williams EH, Smirnoff N, et al (1999) Genetic evidence for the role of GDP-mannose in plant ascorbic acid (vitamin C) biosynthesis. Proc Natl Acad Sci USA 96: 4198–4203. 10097187 10.1073/pnas.96.7.4198 PMC22444

[11] Keller R, Renz FS, Kossmann J (1999) Antisense inhibition of the GDP-mannose pyrophosphorylase reduces the ascorbate content in transgenic plants leading to developmental changes during senescence. Plant J 19: 131–141. 10476060 10.1046/j.1365-313x.1999.00507.x

[12] Badejo AA, Jeong ST, Goto-Yamamoto N, Esaka M (2007) Cloning and expression of GDP-D-mannose pyrophosphorylase gene and ascorbic acid content of acerola (*Malpighia glabra L.*) fruit at ripening stages. Plant Physiol Biochem 45: 665–672. 17764967 10.1016/j.plaphy.2007.07.003

[13] Badejo AA, Tanaka N, Esaka M (2008) Analysis of GDP-D-mannose pyrophosphorylase gene promoter from acerola (*Malpighia glabra*) and increase in ascorbate content of transgenic tobacco expressing the acerola gene. Plant Cell Physiol 49: 126–132. 18037674 10.1093/pcp/pcm164

[14] Badejo AA, Wada K, Gao Y, Maruta T, Sawa Y, et al (2011) Translocation and the alternative D-galacturonate pathway contribute to increasing the ascorbate level in ripening tomato fruits together with the D-mannose/L-galactose pathway. J Exp Bot 63: 229–239. 21984649 10.1093/jxb/err275 PMC3245467

[15] Di Matteo A, Sacco A, Anacleria M, Pezzotti M, Delledonne M, et al (2010) The ascorbic acid content of tomato fruits is associated with the expression of genes involved in pectin degradation. BMC Plant Biol 10: 163–173. 20691085 10.1186/1471-2229-10-163 PMC3095297

[16] Ioannidi E, Kalamaki MS, Engineer C, Pateraki I, Alexandrou D, et al (2009) Expression profiling of ascorbic acid-related genes during tomato fruit development and ripening and in response to stress conditions. J Exp Bot 60: 663–678. 19129160 10.1093/jxb/ern322 PMC2651456

[17] Massot C, Stevens R, Génard M, Longuenesse JJ, Gautier H (2012) Light affects ascorbate content and ascorbate-related gene expression in tomato leaves more than in fruits. Planta 235: 153–163. 21861113 10.1007/s00425-011-1493-x

[18] Stevens R, Buret M, Duffé P, Garchery C, Baldet P, et al (2007) Candidate genes and quantitative trait loci affecting fruit ascorbic acid content in three tomato populations. Plant Physiol 143: 1943–1953. 17277090 10.1104/pp.106.091413 PMC1851805

[19] Stevens R, Page D, Gouble B, Garchery C, Zamir D, et al (2008) Tomato fruit ascorbic acid content is linked with monodehydroascorbate reductase activity and tolerance to chilling stress. Plant Cell Environ 31: 1086–1096. 18433441 10.1111/j.1365-3040.2008.01824.x

[20] Alhagdow M, Mounet F, Gilbert L, Nunes-Nesi A, Garcia V, et al (2007) Silencing of the mitochondrial ascorbate synthesizing enzyme L-galactono-1, 4-lactone dehydrogenase affects plant and fruit development in tomato. Plant Physiol 145: 1408–1422. 17921340 10.1104/pp.107.106500 PMC2151702

[21] Gilbert L, Alhagdow M, Nunes-Nesi A, Quemener B, Guillon F, et al (2009) GDP-D-mannose 3, 5-epimerase (GME) plays a key role at the intersection of ascorbate and non cellulosic cell wall biosynthesis in tomato. Plant J 60: 499–508. 19619161 10.1111/j.1365-313X.2009.03972.x

[22] Zhang C, Liu J, Zhang Y, Cai X, Gong P, et al (2011) Overexpression of *SlGMEs* leads to ascorbate accumulation with enhanced oxidative stress, cold, and salt tolerance in tomato. Plant Cell Rep 30: 389–398. 20981454 10.1007/s00299-010-0939-0

[23] Zou L, Li H, Ouyang B, Zhang J, Ye Z (2006) Cloning, expression, and mapping of GDP-D-mannose pyrophosphorylase cDNA from tomato (*Lycopersicon esculentum*). Acta Genet Sinica 33: 757–764. 10.1016/S0379-4172(06)60108-X 16939010

[24] Zou L, Li H, Ouyang B, Zhang J, Ye Z (2006) Cloning and mapping of genes involved in tomato ascorbic acid biosynthesis and metabolism. Plant Sci 170: 120–127.

[25] Wang HS, Yu C, Zhu ZJ, Yu XC (2011) Overexpression in tobacco of a tomato GMPase gene improves tolerance to both low and high temperature stress by enhancing antioxidation capacity. Plant Cell Rep 30: 1029–1040. 21287174 10.1007/s00299-011-1009-y

[26] Fillatti JAJ, Kiser J, Rose R, Comai L (1987) Efficient transfer of a glyphosate tolerance gene into tomato using a binary *Agrobacterium tumefaciens* vector. Nat Biotechnol 5: 726–730.

[27] Rizzolo A, Forni E, Polesello A (1984) HPLC assay of ascorbic acid in fresh and processed fruit and vegetables. Food Chem 14: 189–199.

[28] Wellburn R (1994) The spectral determination of chlorophylls a and b, as well as total carotenoids, using various solvents with spectrophotometers of different resolution. J Plant Physiol 144: 307–313.

[29] Heath RL, Packer L (1968) Photoperoxidation in isolated chloroplasts.I. Kinetics and stoichiometry of fatty acid peroxidation. Arch Biochem Biophys 125: 189–198. 5655425 10.1016/0003-9861(68)90654-1

[30] Bowling SA, Clarke JD, Liu Y, Klessig DF, Dong X (1997) The *cpr5* mutant of Arabidopsis expresses both NPR1-dependent and NPR1-independent resistance. Plant Cell 9: 1573–1584. 9338960 10.1105/tpc.9.9.1573 PMC157034

[31] Thordal-Christensen H, Zhang Z, Wei Y, Collinge DB (1997) Subcellular localization of H_2_O_2_ in plants. H_2_O_2_ accumulation in papillae and hypersensitive response during the barley-powdery mildew interaction. Plant J 11: 1187–1194.

[32] Tornero P, Gadea J, Conejero V, Vera P (1997) Two *PR-1* genes from tomato are differentially regulated and reveal a novel mode of expression for a pathogenesis-related gene during the hypersensitive response and development. Mol Plant Microbe Interact 10: 624–634. 9204567 10.1094/MPMI.1997.10.5.624

[33] Linthorst HJ, Danhash N, Brederode FT, Van Kan JA, De Wit PJ, et al (1991) Tobacco and tomato PR proteins homologous to *win* and pro-hevein lack the “hevein” domain. Mol Plant Microbe Interact 4: 586–592. 1804403 10.1094/mpmi-4-586

[34] Joosten MH, Bergmans CJ, Meulenhoff EJ, Cornelissen BJ, De Wit PJ (1990) Purification and serological characterization of three basic 15-kilodalton pathogenesis-related proteins from tomato. Plant Physiol 94: 585–591. 16667752 10.1104/pp.94.2.585 PMC1077272

[35] van Kan JA, Joosten MH, Wagemakers CA, van den Berg-Velthuis GC, de Wit PJ (1992) Differential accumulation of mRNAs encoding extracellular and intracellular PR proteins in tomato induced by virulent and avirulent races of Cladosporium fulvum. Plant Mol Biol 20: 513–527. 1421154 10.1007/BF00040610

[36] Cronje C, George GM, Fernie AR, Bekker J, Kossmann J, et al (2012) Manipulation of L-ascorbic acid biosynthesis pathways in *Solanum lycopersicum*: elevated GDP-mannose pyrophosphorylase activity enhances L-ascorbate levels in red fruit. Planta 235: 553–564. 21979413 10.1007/s00425-011-1525-6

[37] Bulley SM, Rassam M, Hoser D, Otto W, Schünemann N, et al (2009) Gene expression studies in kiwifruit and gene over-expression in Arabidopsis indicates that GDP-L-galactose guanyltransferase is a major control point of vitamin C biosynthesis. J Exp Bot 60: 765–778. 19129165 10.1093/jxb/ern327 PMC2652059

[38] Laing WA, Wright MA, Cooney J, Bulley SM (2007) The missing step of the L-galactose pathway of ascorbate biosynthesis in plants, an L-galactose guanyltransferase, increases leaf ascorbate content. Proc Natl Acad Sci USA 104: 9534–9539. 17485667 10.1073/pnas.0701625104 PMC1866185

[39] Bulley S, Wright M, Rommens C, Yan H, Rassam M, et al (2012) Enhancing ascorbate in fruits and tubers through over-expression of the L-galactose pathway gene GDP-L-galactose phosphorylase. Plant Biotechnol J 10: 390–397. 22129455 10.1111/j.1467-7652.2011.00668.x

[40] Pavet V, Olmos E, Kiddle G, Mowla S, Kumar S, et al (2005) Ascorbic acid deficiency activates cell death and disease resistance responses in Arabidopsis. Plant Physiol 139: 1291–1303. 16244149 10.1104/pp.105.067686 PMC1283766

[41] Veljovic-Jovanovic SD, Pignocchi C, Noctor G, Foyer CH (2001) Low ascorbic acid in the *vtc-1* mutant of Arabidopsis is associated with decreased growth and intracellular redistribution of the antioxidant system. Plant Physiol 127: 426–435. 11598218 PMC125079

[42] Takahashi H, Chen Z, Du H, Liu Y, Klessig DF (1997) Development of necrosis and activation of disease resistance in transgenic tobacco plants with severely reduced catalase levels. Plant J 11: 993–1005. 9193071 10.1046/j.1365-313x.1997.11050993.x

[43] Weymann K, Hunt M, Uknes S, Neuenschwander U, Lawton K, et al (1995) Suppression and restoration of lesion formation in Arabidopsis *lsd* mutants. Plant Cell 7: 2013–2022. 12242366 10.1105/tpc.7.12.2013 PMC161058

[44] Greenberg JT, Guo A, Klessig DF, Ausubel FM (1994) Programmed cell death in plants: a pathogen-triggered response activated coordinately with multiple defense functions. Cell 77: 551–563. 8187175 10.1016/0092-8674(94)90217-8

[45] Chamnongpol S, Willekens H, Moeder W, Langebartels C, Sandermann H, et al (1998) Defense activation and enhanced pathogen tolerance induced by H_2_O_2_ in transgenic tobacco. Proc Natl Acad Sci USA 95: 5818–5823. 9576968 10.1073/pnas.95.10.5818 PMC20463

[46] Anderson JC, Pascuzzi PE, Xiao FM, Sessa G, Martin GB (2006) Host-mediated phosphorylation of type ? effector AvrPto promotes *Pseudomonas* virulence and avirulence in tomato. Plant Cell 18: 502–514. 16399801 10.1105/tpc.105.036590 PMC1356555

[47] Müller-Moulé P, Golan T, Niyogi KK (2004) Ascorbate-deficient mutants of Arabidopsis grow in high light despite chronic photooxidative stress. Plant Physiol 134: 1163–1172. 14963245 10.1104/pp.103.032375 PMC389940

[48] Pastori GM, Kiddle G, Antoniw J, Bernard S, Veljovic-Jovanovic S, et al (2003) Leaf vitamin C contents modulate plant defense transcripts and regulate genes that control development through hormone signaling. Plant Cell 15: 939–951. 12671089 10.1105/tpc.010538 PMC152340

[49] Kiddle G, Pastori GM, Bernard S, Pignocchi C, Antoniw J, et al (2003) Effects of leaf ascorbate content on defense and photosynthesis gene expression in *Arabidopsis thaliana* . Antioxid Redox Signal 5: 23–32. 12626114 10.1089/152308603321223513

