# Supplementary figures and images for: Reducing AsA Leads to Leaf Lesion and Defence Response in Knock-Down of the AsA Biosynthetic Enzyme GDP-D-Mannose Pyrophosphorylase Gene in Tomato Plant
Source: PLoS One. 2013 Apr 23;8(4):e61987. doi: 10.1371/journal.pone.0061987 (PMC3633959; doi:10.1371/journal.pone.0061987)

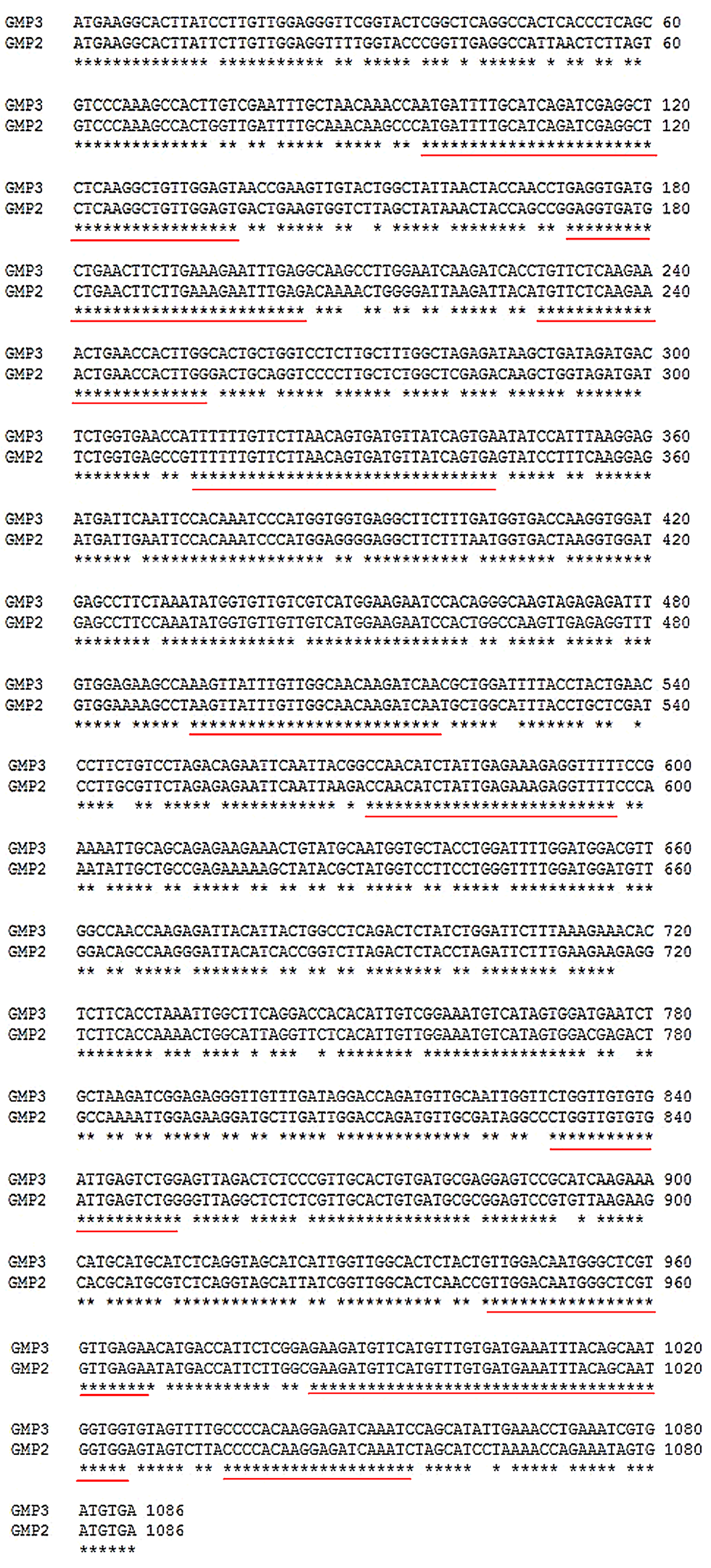

Supplement: Figure S1 — Nucleotide sequence alignment of SlGMP2 and SlGMP3 genes. (TIF) [file pone.0061987.s001.tif]
