# Supplementary material for: Reducing AsA Leads to Leaf Lesion and Defence Response in Knock-Down of the AsA Biosynthetic Enzyme GDP-D-Mannose Pyrophosphorylase Gene in Tomato Plant
Source: PLoS One. 2013 Apr 23;8(4):e61987. doi: 10.1371/journal.pone.0061987 (PMC3633959; doi:10.1371/journal.pone.0061987)
Supplement: Table S1 — Primers used for SlGMP3 amplification and identification of the transformants and RT-PCR. (DOC) [file pone.0061987.s002.doc]

**Table S1. Primers used for *SlGMP3* amplification and identification of transformants and RT-PCR.**

| **Primer names** | **Sequences (5′–3′)** |
| --- | --- |
| GMP3F | ATTCAGTCTTTGGATTTTCTCG |
| GMP3R | GAAGAAGAGGAGAACTGGAAAC |
| β-actinF | ATGGCAGACGGAGAGGATATTCA |
| β-actinR | GCCTTTGCAATCCACATCTGCTG |
| CaMV35S | ACGCACAATCCCACTATCCTTC |
| gate35S | CGTAAGGGATGACGCACAA |
