# Supplementary material for: Reducing AsA Leads to Leaf Lesion and Defence Response in Knock-Down of the AsA Biosynthetic Enzyme GDP-D-Mannose Pyrophosphorylase Gene in Tomato Plant
Source: PLoS One. 2013 Apr 23;8(4):e61987. doi: 10.1371/journal.pone.0061987 (PMC3633959; doi:10.1371/journal.pone.0061987)
Supplement: Table S2 — Primers used for real-time RT-PCR of the AsA biosynthesis-related genes. (DOC) [file pone.0061987.s003.doc]

**Table S2. Primers used for real-time RT-PCR of the AsA biosynthesis-related genes.**

| **Gene** | **Forward primer (5′–3′)** | **Reverse primer (5′–3′)** | **Accession No.** | **Size (bp)** |
| --- | --- | --- | --- | --- |
| *GPI* | TGCTCTTCAAAAGCGTGTCC | CGGCAATAAGTGCTCTGTCA | SGN-U568685 | 170 |
| *PMI* | TACATTGTGGTGGAACGAGGA | ACCCCATTTGGCAAGAACAG | SGN-U576695 | 138 |
| *PMM* | TTTACCCTCCATTACATTGCTGA | TCTTCTTGACTACAGTTTCTCCCA | SGN-U573853 | 116 |
| *GMP1* | TGAAATCAAGGAAAATGCGG | ACCGACACGGATTCACCAAG | SGN-U563807 | 138 |
| *GMP2* | GCATTATCGGTTGGCACTCA | GGGTAAGACTACTCCACCATTGC | SGN-U568548 | 119 |
| *GMP3* | AAACCTGAAATCGTGATGTGAGA | TGAAGAAGAGGAGAACTGGAAAC | SGN-U568547 | 124 |
| *GMP4* | AGACAGAGCCTTTAGGCACAGC | GCCGTGGGACTTATGAAACAA | SGN-U584300 | 149 |
| *GME1* | AATCCGACTTCCGTGAGCC | CTGAGTTGCGACCACGGAC | SGN-U581327 | 147 |
| *GME2* | CCATCACATTCCAGGACCAGA | CGTAATCCTCAACCCATCCTT | SGN-U580326 | 115 |
| *GGP1* | GAAATCTGGTCTGTTCCTCTGTGA | TTCACACACCAACTCCACATTACA | SGN-U579800 | 140 |
| *GGP2* | CTGTTGTCTTGGTTGGAGGTTGT | AGCACAGTCAAAACACCAACAAA | SGN-U573852 | 102 |
| *GP1* | AGCCGCTACAAACCCTCATCT | TGTCCGCTTTCCATCTCCTAT | SGN-U600217 | 87 |
| *GP2* | GGTTAGGTCCCTTCGTATGTG | TTTCACAATCACAGCACCACC | SGN-U568299 | 133 |
| *GalDH* | CTTCTTACTGAGGCTGGTGGTC | AACCTCTTTAACAGACTTCATCCC | SGN-U565558 | 186 |
| *GLDH* | ATTGAGGTTCCCAAGGACATAG | ATGTTATTAGATAGGATGCGGTTT | AB080690 | 128 |
| *Actin* | GTCCTCTTCCAGCCATCCA | ACCACTGAGCACAATGTTACCG | BT013524 | 126 |
