# Supplementary material for: Reducing AsA Leads to Leaf Lesion and Defence Response in Knock-Down of the AsA Biosynthetic Enzyme GDP-D-Mannose Pyrophosphorylase Gene in Tomato Plant
Source: PLoS One. 2013 Apr 23;8(4):e61987. doi: 10.1371/journal.pone.0061987 (PMC3633959; doi:10.1371/journal.pone.0061987)
Supplement: Table S3 — Primers used for real-time RT-PCR of the photosynthesis-related genes. (DOC) [file pone.0061987.s004.doc]

**Table S3. Primers used for real-time RT-PCR of the photosynthesis-related genes.**

| **Gene** | **Forward primer (5′–3′)** | **Reverse primer (5′–3′)** | **Accession No.** | **Size (bp)** |
| --- | --- | --- | --- | --- |
| *Cab1B* | ATGGGCTATGCTTGGTGCTC | TCGCTGAAAATCTGGGAACC | SGN-U580483 | 81 |
| *Cab3B* | ATGAGGAAGACTGCCGCTG | CAGCAGTGTCCCATCCGTAG | SGN-U581613 | 89 |
| *Cab3C* | CCGGTAAGGCGGTGAAACT | CAGGACCGTACCATGGGCTA | [SGN-U592308](http://solgenomics.net/search/unigene.pl?unigene_id=592308) | 70 |
| *Cab-4* | GCTTGACTACCTTGGAAACCC | CACCTCCTGGGTAGATCTTGTCA | SGN-U579113 | 78 |
| *LHCA5* | TTGAGTTTGCTTGTTGAGTGGTG | GCCAGAGGTGTTAGAGAATGTCC | [SGN-U577781](http://solgenomics.net/search/unigene.pl?unigene_id=577781) | 73 |
| *TK* | AGAAGACGGTCCAACTCATCAAC | TCCTGCTGTCTCATTACCATCTG | [SGN-U578988](http://solgenomics.net/search/unigene.pl?unigene_id=578988) | 73 |
| *ATPs* | TCGTTTCATCTACAGGCATTCA | GCAAGTTCAGTATCCGTCAAGG | [SGN-U583491](http://solgenomics.net/search/unigene.pl?unigene_id=583491) | 100 |
| *PhyB1* | AACTCCCTGACACTCTCAACAACA | GAGGACCCTGCTTTATCTATTGC | [SGN-U583481](http://solgenomics.net/search/unigene.pl?unigene_id=583481) | 94 |
| *PSI-N* | AGAAGAAATGGCAGCAATGAAC | TTTTGAAGTGAAGGGGAAGACA | [SGN-U577591](http://solgenomics.net/search/unigene.pl?unigene_id=577591) | 88 |

Cab: chlorophyll *a*-*b* binding protein, LHCA: photosystem I light harvesting complex gene, TK: transketolase, chloroplast precursor, ATPs: ATP synthase delta chain, chloroplastic, PhyB1: phytochrome B1, and PSI-N: photosystem I-N subunit.
