# Supplementary material for: Reducing AsA Leads to Leaf Lesion and Defence Response in Knock-Down of the AsA Biosynthetic Enzyme GDP-D-Mannose Pyrophosphorylase Gene in Tomato Plant
Source: PLoS One. 2013 Apr 23;8(4):e61987. doi: 10.1371/journal.pone.0061987 (PMC3633959; doi:10.1371/journal.pone.0061987)
Supplement: Table S4 — Primers used for real-time RT-PCR of the pathogenesis-related genes and antioxidant enzyme genes. (DOC) [file pone.0061987.s005.doc]

**Table S4. Primers used for real-time RT-PCR of the pathogenesis-related genes and antioxidant enzyme genes.**

| **Gene** | **Forward primer (5′–3′)** | **Reverse primer (5′–3′)** | **Accession No.** | **Size (bp)** |
| --- | --- | --- | --- | --- |
| *PR1b1* | GCACTAAACCTAAAGAAAAATGGG | GCATCGTTATGAACCGCAAG | Y08804 | 132 |
| *PR-P2* | GGAACAGGAACACAAGAAACAGTGA | CCCAATCCATTAGTGTCCAATCG | X58548 | 104 |
| *PR-P6* | AGTCGGAGTCGGGCCTATGT | TCTCCCCAGCACCAGAATGA | M69248 | 110 |
| *CAT* | AAGTCCTGTGGTCAGAAGGTCG | GAAGTACAGTTTATAGCACAACGCG | SGN-U578839 | 164 |
| *SOD* | TGAATTGGGGTTGAACCATT | GCAGGCACTGTAATCTGCAA | SGN-U581604 | 188 |
| *cAPX* | ACGATGATATTGTGACACTCTTCCA | AAGCGATGAAACCACAAAAACA | DQ099420 | 71 |
| *ChlAPX1* | CATGCCAAACTGAGCAACCTT | CCTCCCAAGCCTTCGTATTCT | DQ131132 | 179 |
| *ChlAPX2* | CTTTCTTCAATGGCTTCTCTCACCG | CAACCTGGTAGCGAAACACATGGG | DQ131133 | 165 |

PR: pathogenesis related, CAT: catalase, SOD: superoxide dismutase, cAPX: cytosolic ascorbate peroxidase, and ChlAPX: chloroplastic ascorbate peroxidase.
