# Supplementary material for: Reducing AsA Leads to Leaf Lesion and Defence Response in Knock-Down of the AsA Biosynthetic Enzyme GDP-D-Mannose Pyrophosphorylase Gene in Tomato Plant
Source: PLoS One. 2013 Apr 23;8(4):e61987. doi: 10.1371/journal.pone.0061987 (PMC3633959; doi:10.1371/journal.pone.0061987)
Supplement: Table S5 — The fruit weights and yields of SlGMP2/3 -KD lines and wild-type plants. (DOC) [file pone.0061987.s006.doc]

**Table S5. The fruit weights and yields of *SlGMP2/3*-KD lines and wild-type plants.**

|  | **WT** | **KD7** | **KD17** |
| --- | --- | --- | --- |
| **Fruit weight (g)** | 22.68 ± 1.7 | 18.07 ± 0.69 * | 23.98 ± 1.27 |
| **Yield (g)** | 494.98 ± 51.77 | 384.37 ± 42.53 | 454.14 ± 37.95 |

Data presented as Mean ± SD of five independent plants per line. * indicates values that are significantly different from those of wild-type plants (*P* > 0.95).
